# Giant Soft Tissue Leiomyosarcoma of the Left Lower Extremity: Case Presentation With a Review of the Literature

**DOI:** 10.7759/cureus.37058

**Published:** 2023-04-03

**Authors:** Giovanni De Biasi, Gerardo Cazzato, Anna Colagrande, Eugenio Maiorano, Giuseppe Ingravallo

**Affiliations:** 1 Department of Precision and Regenerative Medicine and Ionian Area (DiMePRe-J) Section of Molecular Pathology, University of Bari "Aldo Moro", Bari, ITA; 2 Department of Emergency and Organ Transplantation, University of Bari "Aldo Moro", Bari, ITA; 3 Department of Emergency and Organ Transplantation, Section of Pathology, University of Bari "Aldo Moro", Bari, ITA

**Keywords:** bone, oncology soft tissue, trauma and orthopedics, pathology, soft tissue tumours

## Abstract

Leiomyosarcoma (LMS) accounts for approximately 5-10% of soft tissue sarcomas, with an estimated incidence in the United States (US) of less than one case/200,000 persons, more frequent in women than men. Approximately two-thirds of LMSs are retroperitoneal, abdominal, and mediastinal. Localized, soft tissue LMSs represent a lower percentage, with the lower limbs and trunk being the most frequently involved sites. LMSs larger than 5 cm (so-called giants) are even rarer, and to date have been little reported in the literature. In this paper, we present the case of a giant LMS of the left lower limb in a 73-year-old patient, who had a mass for about two years, and who, after the first diagnostic biopsy, underwent limb amputation. Macroscopic and microscopic examinations confirmed the infiltration of the underlying tibial bone. We briefly discuss eight other cases described in the literature with similar size, pointing out that the parameters with the greatest impact on prognosis proved to be size >5 cm and depth of invasion. Due to the rarity of this neoplasm, little has yet been done in relation to the most suitable therapeutic treatment of such patients, and larger case series are mandated in order to be able to conduct broader-spectrum studies.

## Introduction

Leiomyosarcoma (LMS) accounts for 5-10% of soft tissue sarcomas, with an incidence in the United States (US) of less than 1 case/200,000 people [1-2]. Although LMSs are most frequent in the retroperitoneum, abdominal cavity, and mediastinum [3], cases have been reported in peripheral locations, with the lower limbs being the most frequent site of this subtype of LMS [4]. Topographically, soft tissue LMS are divided into cutaneous (dermal) and subcutaneous, and compared with retroperitoneal lesions, soft tissue LMS of the extremities and trunk are much less common and affect the sexes equally [1,5]; furthermore, even rarer are LMSs larger than 5 cm, with only eight cases reported in the English literature. Due to the low incidence and the consequent small number of large case series in the literature, there are still many question marks regarding the best surgical and/or oncological treatment, as well as difficulties in implementing innovative therapies such as immunotherapy and gene therapy [6]. In addition, the question of the precise origin of some advanced forms of LMS remains open, since as it is generally not recognized early, it is almost impossible to establish the origin from potential smooth muscle cells of the vessels and/or the cutaneous piloerector muscle [7]. Here, we report a case of a giant subcutaneous soft tissue LMS arising in the left lower limb of a 73-year-old patient and briefly discuss other similar cases reported in the current literature.

## Case presentation

**Figure 1:**
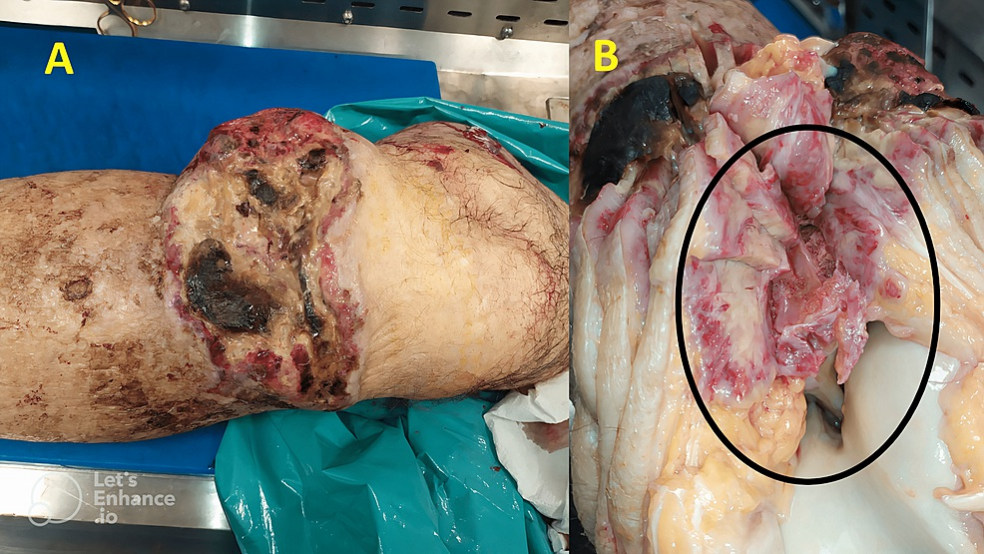
(A) Left lower limb surgical specimen, including skin, subcutis, fascia, muscle, and bone of the distal end of the femur, tibia, fibula, and left foot (not shown). On macroscopic observation, at the proximal third of the leg, a large, superficially ulcerated and bleeding neoformation, measuring 17 x 9 cm, was found, which macroscopically appeared to infiltrate the tibial bone plane (B, black circle).

The patient, who was in good health, complained of a subcutaneous swelling of the proximal third of his left leg in the post-traumatic setting. Following an orthopedic consultation, an initial biopsy was decided upon for nosographic diagnostic framing. The first histological report described a malignant mesenchymal neoplasm consisting of spindle cells, organized in bundles and focally pleomorphic; there were large areas of coagulative necrosis and aspects of fibrosclerosis. On this biopsy, a mitotic index of 5 mitoses/mm^2^ was described and a diagnosis of Grade II leiomyosarcoma according to the Fédération Nationale des Centres de Lutte Contre le Cancer (FNCLCC) was made. Two months later, the patient was referred for amputation of the distal portion of the lower limb, and the surgical specimen was sent to the pathology department. On macroscopic examination (Figure 1A), a centrally ulcerated lesion was found, measuring 17 x 9 cm, with extensive necrosis and doubtful infiltration at the anterolateral surface of the left tibia (Figure 1B).

**Figure 2:**
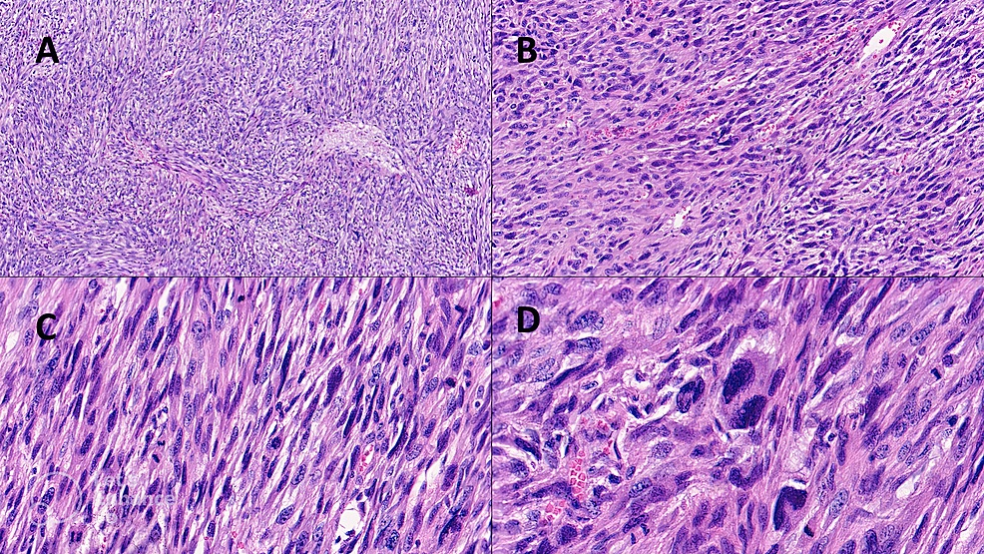
(A) Histological photomicrograph showing LMS constituted by elongated, plump neoplastic cells arranged in fascicles of varying sizes, with areas of right angles intersection (more differentiated areas) (Hematoxylin-Eosin stain, original magnification 2x). (B) Area of LMS characterized by blunt-ended nuclei and eosinophilic cytoplasm. (Hematoxylin-Eosin stain, original magnification 4x). (C) Microscopic picture shows the pleomorphic area of LMS with some typical and atypical mitoses, cells with larger and hyperchromatic nuclei, sometimes with perinuclear vacuoles, and many vessels. (Hematoxylin-Eosin stain, original magnification 10x). (D) Details of some giant cells with hyperchromatic nuclei and numerous mitoses. (Hematoxylin-Eosin stain, original Magnification 20x). LMS: Leiomyosarcoma

On microscopic examination, the same features as in the first biopsy were described, with a histological staging of grade 3 according to the FNCLCC (Fédération Nationale des Centres de Lutte Contre le Cancer) system (Tumor Differentiation: score 2; Mitotic Count: score 3: >/=20 mitoses/10 High Power Field (HPF); Tumor Necrosis (microscopic): >/=50%) (Figures 2A-2D).

**Figure 3:**
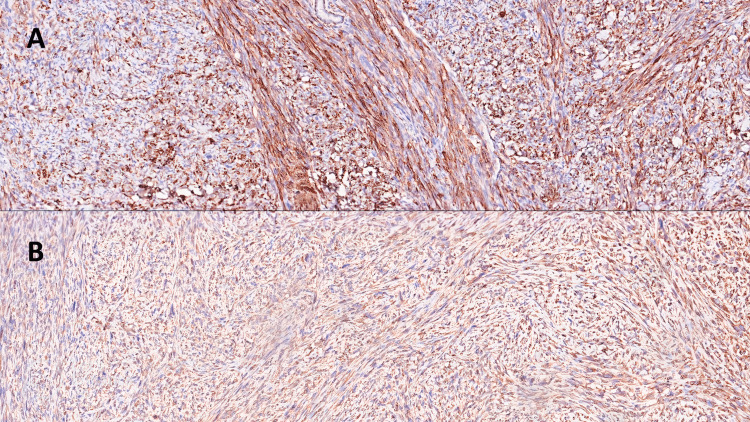
(A) Immunohistochemical preparation for smooth muscle actin (SMA): note the diffuse positivity of the neoplastic cells, with different types of spatial orientation. (Immunohistochemistry for SMA, Original Magnification 4x). (B) Immunohistochemical preparation for vimentin: note, also in this case, the diffuse positivity of the neoplastic cells. (Immunohistochemistry for vimentin, Original Magnification 4x).

Immunohistochemical staining was strongly positive for smooth muscle actin (SMA) and vimentin (Figures 3A-3B) while negative for actin HHF-35, sarcomeric actin, h-caldesmon, and desmin (not shown).

With the combination of the histological and immunohistochemical picture, a diagnosis of LMS grade III according to FNCLCC was made, and at the three-month follow-up, the patient did not appear to present a disease recurrence.

## Discussion

Soft tissue LMS represents a rarer entity than its retroperitoneal/abdominal counterpart and, by virtue of anatomical localization, tends to grow smaller in size [1,6]. For these reasons, cases of giant soft tissue LMSs are rather rare, and after a brief review of the literature, to the best of our knowledge, only eight cases have been described to date [8-15].

Table 1 summarizes the cases described in the literature to date, with emphasis on gender, age, location, histological diagnosis and relative grading of the neoplasm, and information (when available) on patient follow-up.

**Table 1:** Features of the giant LMSs described in the literature LMS: Leiomyosarcoma; NR: No relevant; SMA: Smooth muscle actin; IHC: Immunohistochemistry

Authors	Jena et al. [15]	Angeloni et al. [14]	Talikoti et al. [13]	Eken et al. [12]	Palla et al. [11]	Chuanping et al. [10]	Rizwan et al. [9]	Yajima et al. [8]
gender	female	male	male	male	female	male	male	male
age	35	71	67	44	81	29	65	31
topography	right abdomen	right tibia	scrotum	abdomen wall	shoulder	thigh	thigh	inguinal
dimensions (cm)	20x20	12x10	28x25x15	26×24×15	20	9x5,1x7,2	24x17x9	12,4x10,5
time to diagnosis/growth	2 years	slow growth (no time)	history (40 years) of filariasis	3 years	slow growth (no time)	6 months (first recurrence)	6 months	6 months
histological examination	FNCLCC Grado II (2+2+0)	Broders Grade III	no grading	no grading	pT2 NCI:1+	no grading	FNCLCC Grade III	no grading
surgical outcomes	NR 4 months	NR 3 years (CT+RT)	NR 19 months	NR 3 years	no FU	NR 1 year	recovery in 2 months	NR 5 years and 8 months
IHC	SMA+ CD34+, Desmina- S100- Ki67 10-15%	SMA+ Vimentin+ Caldesmon+	SMA+, Vimentin+, Desmin+, S100-	SMA+ Vimentin+ Desmin+, CD117- S100-	no IHC	SMA+ Desmin+	SMA+	SMA+ desmin+, vimentin-, S100-

Of the eight cases reported in the literature, two cases involved the abdominal wall/abdomen [12,15], with very large dimensions of no less than 20 cm in maximum diameter. Conversely, Rizwan T and Chuanping G described two LMSs at the level of the thigh, with a maximum diameter of 24 cm and 9 cm, respectively [9,10]. Our case is quite similar to the one reported by Angeloni et al., in which a lesion at the right tibia measuring 12 x 10 cm was reported. In all eight cases reported, the follow-up reports available up to the time of registration reported no recurrence of disease, a finding quite consistent with those reported in the Scandinavian series [16] in which 84% of patients with localized disease at presentation remained free of recurrent disease at a median follow-up of 5.5 years, with distant metastases occurring in 34% of cases and death in 51% of patients. In the most recent French series, the authors reported that approximately 40% of patients with soft tissue LMSs were metastasis-free at a follow-up of 140 months, in contrast to the rates of 75%, 60%, and 25% of patients with LMSs of the trunk, head/neck and retroperitoneum [17].

Furthermore, it is clear from the case reports that the prognostic factors most indicative of an adverse prognosis are the size of the LMS (>5 cm) and the depth of invasion [8-15]. It is rather intuitive to say that such situations as the one in the case we presented constitute a 'defeat' of preventive/early intervention medicine, as the possibility of loco-regional and metastatic spread is high, despite the amputation of the limb performed.

The main differential diagnoses of LMS are both non-pleomorphic spindle cell tumors and other types of pleomorphic sarcomas [1]. Primarily, it is necessary to differentiate LMS from gynecological and non-gynecological smooth muscle leiomyomas, paying particular attention to the fact that leiomyomas are characterized by thick-walled blood vessels, solid, trabecular, and cord-like growth patterns, with the absence or focal presence of cytological atypical mitoses [1,18-19]. Cellular schwannomas are benign encapsulated, cellular, fasciculated lesions consisting of Schwann cells that are positive for S-100 protein and negative (usually) for actin and desmin. On the other hand, the differential diagnosis with gastrointestinal stromal tumors (GISTs) is performed by immunohistochemical staining for CD117 (c-kit) and DOG1 (positive in >90% of GISTs) [18]. An inflammatory myofibroblastic tumor usually arises in younger individuals than true muscle lesions and is characterized by the presence of inflammatory infiltration sometimes associated with calcifications [19]. The differential diagnosis is even easier with monophasic synovial sarcoma, both from a morphological and immunophenotypic point of view and with malignant peripheral nerve sheath tumor (MPNST), which tends to lose diffuse cytoplasmic eosinophilia typical of LMS and shows focal positivity for S-100 and/or SOX-10 but not for actin and/or desmin [1].

Despite progress and improved therapies, surgery remains the 'gold standard' for cases such as those reported. In particular, some authors have suggested the use of radiotherapy to eradicate any potential residual neoplasm [9] while chemotherapy (CHT) is used in the case of metastatic/recurrent disease [18-19].

Further cases with clinicopathological correlations and more case series are mandated to obtain more information on the best diagnostic, therapeutic, and care management of the patient with a rare neoplasm such as soft tissue LMS.

## Conclusions

Soft tissue leiomyosarcoma, being rarer than its retroperitoneal/abdominal counterpart, is also less studied, and, therefore, case reports, case series, and original articles investigating this entity are much needed. From the case presented by us, it is quite clear that the latency time can be quite long, but cases of very fast development of LMS have not infrequently been described in the literature. The difficulties concerning the correct determination of the muscular compartment from which the soft tissue LMS may originate are very important to emphasize, although this is not helpful for the treatment or staging of LMS, which remains unavoidable.

## References

[1] Goldblum JR, Weiss SW, Folpe LA. (2020). Leiomyosarcoma. Enzinger and Weiss's Soft Tissue Tumors.

[2] Gustafson P, Willen H, Baldetorp B, Fernö M, Akerman M, Rydholm A. (1992). Soft tissue leiomyosarcoma. A population‐based epidemiologic and prognostic study of 48 patients, including cellular DNA content. Cancer.

[3] Marko J, Wolfman DJ (2018). Retroperitoneal leiomyosarcoma from the radiologic pathology archives. Radiographics.

[4] Lee MS, Shi CR, Sauer M, Laga AC, Talia J, Nambudiri VE (2021). Bilateral lower extremity induration in a patient with leiomyosarcoma. Lancet Oncol.

[5] Cazzato G, Sergi MC, Sablone S (2021). Advanced cutaneous leiomyosarcoma of the forearm. Dermatopathology (Basel).

[6] Devaud N, Vornicova O, Abdul Razak AR (2022). Leiomyosarcoma: current clinical management and future horizons. Surg Oncol Clin N Am.

[7] Farshid G, Pradhan M, Goldblum J, Weiss SW (2002). Leiomyosarcoma of somatic soft tissues: a tumor of vascular origin with multivariate analysis of outcome in 42 cases. Am J Surg Pathol.

[8] Yajima K, Shirai Y, Fujita N, Sato D, Umezu H, Hatakeyama K (2005). A giant subcutaneous leiomyosarcoma arising in the inguinal region. World J Surg Oncol.

[9] Rizwan T, Ahmed J, Shaikh FH, Malik F, Ullah S (2020). Giant leiomyosarcoma arising in posterior thigh: management of a rare case. Cureus.

[10] Chuanping G, Weiwei F (2015). Recurrent, giant subcutaneous leiomyosarcoma of the thigh. Radiol Case Rep.

[11] Palla L, Gentile P, Cannatà C, Ascenzi P, Buonomo O, Cervelli V (2009). A neglected giant subcutaneous leiomyosarcoma of the shoulder: a case report. Eur Rev Med Pharmacol Sci.

[12] Eken H, Karagul S, Topgül K (2016). Giant cutaneous leiomyosarcoma originating from the abdominal wall: a case report. Am J Case Rep.

[13] Talikoti MA, Deo SS, Shukla NK, Kallianpur AA, Gupta M (2011). A rare case of giant leiomyosarcoma in a filarial scrotum: a case report. World J Surg Oncol.

[14] Angeloni M, Muratori F, Magarelli N, Chalidis BE, Ricci R, Rossi B, Maccauro G (2008). Exophytic growth of a neglected giant subcutaneous leiomyosarcoma of the lower extremity. A case report. Int Semin Surg Oncol.

[15] Jena S, Bhattacharya S, Roy S (2014). Giant subcutaneous leiomyosarcoma of anterior abdominal wall. Case Rep Surg.

[16] Svarvar C, Böhling T, Berlin O (2007). Clinical course of nonvisceral soft tissue leiomyosarcoma in 225 patients from the Scandinavian Sarcoma Group. Cancer.

[17] Salas S, Stoeckle E, Collin F (2009). Superficial soft tissue sarcomas (S-STS): a study of 367 patients from the French Sarcoma Group (FSG) database. Eur J Cancer.

[18] Divyambika CV, Sathasivasubramanian S, Krithika CL, Malathi N, Prathiba D (2012). Pediatric oral leiomyosarcoma: rare case report. J Cancer Res Ther.

[19] Guerriero S, Sborgia A, Giancipoli G, Fiore MG, Ross R, Piscitelli D (2011). A rare case of primitive epithelioid leiomyosarcoma of the conjunctiva. Orbit.

